# HCF-1 encoded by baculovirus AcMNPV is required for productive nucleopolyhedrovirus infection of non-permissive Tn368 cells

**DOI:** 10.1038/s41598-017-03710-z

**Published:** 2017-06-19

**Authors:** Ami Tachibana, Rina Hamajima, Moe Tomizaki, Takuya Kondo, Yoshie Nanba, Michihiro Kobayashi, Hayato Yamada, Motoko Ikeda

**Affiliations:** Laboratory of Sericulture and Entomoresources, Graduate School of Bioagricultural Sciences, Nagoya University, Chikusa, Nagoya, 464-8601 Japan

## Abstract

Baculovirus Autographa californica multiple nucleopolyhedrovirus (AcMNPV) replicates in both *Spodoptera frugiperda* Sf21 and *Trichoplusia ni* Tn368 cells, whereas AcMNPV defective in *hcf-1* (host cell-factor 1) gene productively infects only Sf21 cells, indicating that HCF-1 is indispensable for the AcMNPV productive infection of Tn368 cells. Here, we demonstrated that HCF-1 protein transiently expressed in Tn368 cells promotes the DNA synthesis of Hyphantria cunea MNPV ﻿(HycuMNPV), Orygia pseudotsugata MNPV and *Bombyx mori* NPV, which are normally unable to replicate in Tn368 cells. We also demonstrated that a recombinant HycuMNPV harboring the *hcf-1* gene successfully replicates in Tn368 cells, generating substantial yields of progeny viruses and polyhedra. These results indicate that HCF-1 encoded by AcMNPV is an essential viral factor for productive NPV infection of Tn368 cells. Taken together with the previous findings on HRF-1 (host range factor 1), the present results provide strong evidence that viral genes acquired through horizontal gene transfer play an important role in baculovirus evolution, serving to expand the host range of baculoviruses.

## Introduction

Nucleopolyhedroviruses (NPVs), which belong to members of the family *Baculoviridae*, are large, enveloped, insect-pathogenic viruses that contain a double-stranded circular DNA genome of 80 to 180 kbp^1^. NPVs generally exhibit a narrow host range and are capable of productively infecting only one or a few closely related insect species. Notably, however, Autographa californica multiple nucleopolyhedrovirus (AcMNPV), and Anagrapha falcifera and Rachiplusia ou MNPVs, which are close relatives of AcMNPV, are capable of infecting over 30 lepidopteran insect species within 12 families^2–5^.

Several viral genes that determine the host range of NPVs have been identified and include *p143*, *ie-2*, *lef-7* (late expression factor-7), *p35*, *iap*s (inhibitors of apoptosis), host range factor 1 (*hrf-1*), and host cell-specific factor 1 (*hcf-1*)^6–8^. The *p143*, *ie-2*, *lef-7*, and *hcf-1* play a role in viral DNA replication or late gene expression, whereas the *p35* and *iap*s are involved in an antiviral defense system of insects, serving as anti-apoptotic genes. These genes are differentially required for optimal viral replication in a cell-line specific manner. For example, recombinant AcMNPV defective in functional *p35* gene normally replicates in Tn368 cells derived from the cabbage looper *Trichoplusia ni*, but induces massive apoptosis in Sf21 cells derived from the fall armyworm *Spodoptera frugiperda*, yielding little or no progeny viruses^9, 10^. Similarly, AcMNPV harboring *p143* gene derived from Bombyx mori NPV in place of its native *p143* replicates successfully in non-permissive *B*. *mori* BmN-4 or Bm5 cells^11, 12^. Notably, the HRF-1 and HCF-1 have been shown to contribute to AcMNPV replication exclusively in *Lymantria dispar* Ld652Y cells and Tn368 cells, respectively^13–16^. Despite the identification and characterization of these genes, the molecular mechanisms underlying the host range determination of NPVs remain largely elusive.

AcMNPV replicates to high titers in both Sf21 and Tn368 cells. In recombinant AcMNPV defective in the *hcf-1* gene, however, productive infection only occurs in Sf21 cells, indicating that AcMNPV requires HCF-1 for the productive infection of Tn368 cells^14, 15^. The HCF-1 protein contains a putative RING-finger domain, which is involved in the formation of functional HCF-1 dimers or higher-order structures in the nucleus of infected Tn368 cells^17, 18^. It was also demonstrated that transiently expressed HCF-1 protein represses expression from the *hcf-1* promoter and this repression activity of HCF-1 is required for the efficient expression of viral late genes and production of polyhedra in Tn368 cells^18^. However, the functional role of HCF-1 protein in AcMNPV-infected Tn368 cells has not been conclusively determined.

In the present study, we demonstrated that transiently expressed HCF-1 protein promotes the productive infection of non-permissive Tn368 cells by Hyphantria cunea MNPV (HycuMNPV). Recombinant HycuMNPV harboring the *hcf-1* gene also successfully replicated in Tn368 cells, indicating that *hcf-1* gene embedded in the HycuMNPV genome functions effectively for HycuMNPV productive infection of Tn368 cells. In contrast, Orgyia pseudotsugata MNPV (OpMNPV) and Bombyx mori NPV (BmNPV) were capable of viral DNA replication in HCF-1-expressing Tn368 cells, but not the synthesis of viral structural or polyhedral proteins. Taken together, these results indicate that HCF-1 protein is an essential viral factor for the productive NPV infection of Tn368 cells, but is not sufficient to promote viral protein synthesis of certain NPVs in infected Tn368 cells.

## Results

### HCF-1 promotes viral DNA and viral protein production of certain NPVs in non-permissive Tn368 cells

**Figure 1:**
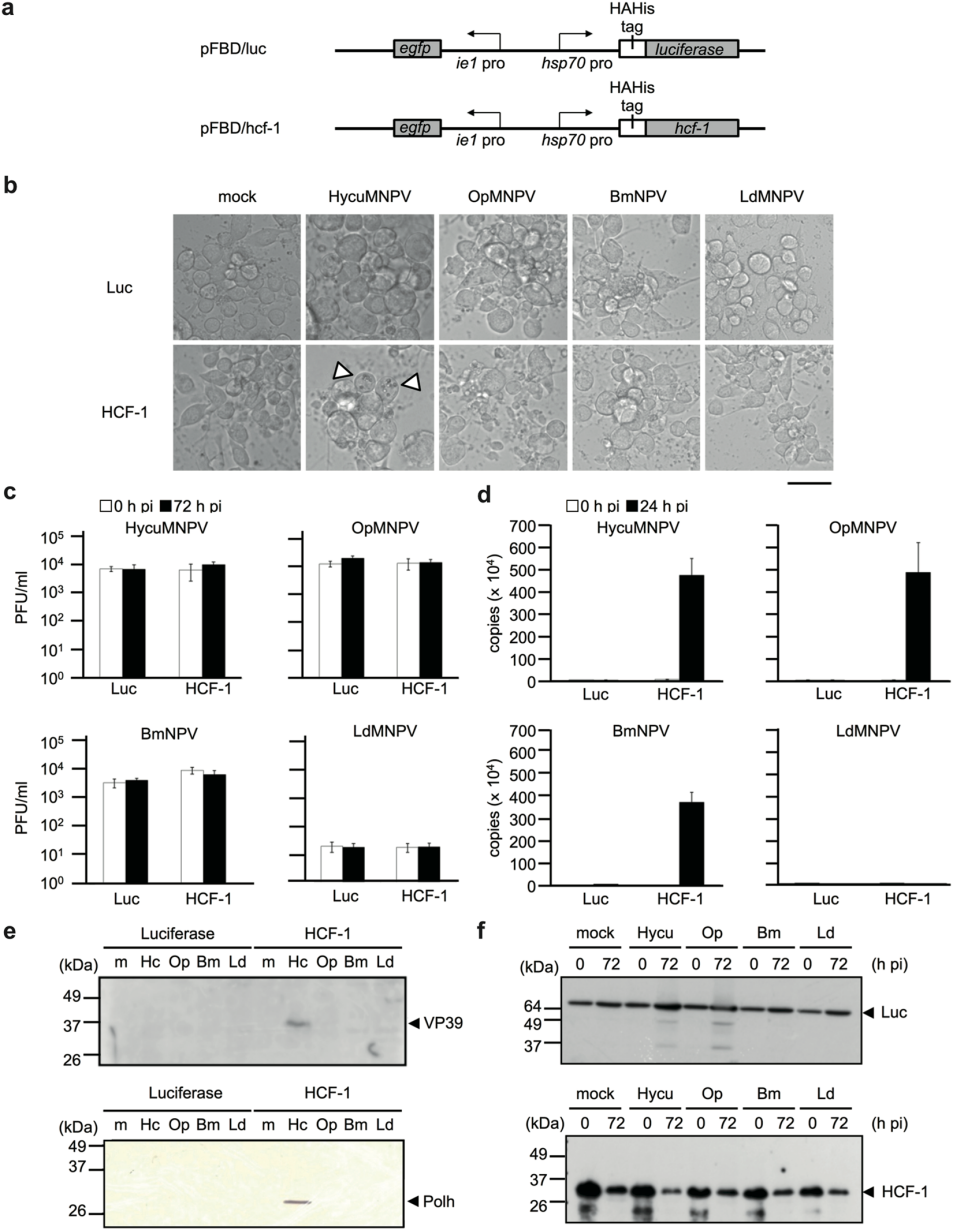
Transiently expressed HCF-1 protein promotes the synthesis of NPV DNA and proteins in non-permissive Tn368 cells. Tn368 cells were transfected with 2 μg of pFBD/luc (Luc) or pFBD/hcf-1 (HCF-1) DNA, which express luciferase or HCF-1 protein, respectively, under control of the heat shock protein 70 promoter (*hsp70* pro). At 24 h post-transfection, the transfected cells were heat-shocked at 42 °C for 30 min, incubated for 6 h at 28 °C, and were then infected with HycuMNPV (Hycu), OpMNPV (Op), BmNPV (Bm) and LdMNPV (Ld) at MOIs of 10, 50, 10 and 3, respectively. (**a**) Schematic representation of the plasmids pFBD/luc and pFBD/hcf-1 used in these experiments. (**b**) Microscopic images of Tn368 cells at 72 h post-infection. The arrowheads indicate polyhedra-containing cells. Scale bar, 50 μm. (**c**) BV yields in virus-infected Tn368 cells at 72 h post-infection (pi). BV titers were determined by a plaque assay using SpIm cells for HycuMNPV, BM-N cells for BmNPV and Ld652Y cells for OpMNPV and LdMNPV. The vertical bars represent the standard deviations of the averages from three determinations. (**d**) Viral DNA production at 24 h post-infection. Viral DNA was quantified by qPCR. The vertical bars represent the standard deviations of the averages from three determinations. (**e**) Immunoblot analysis of VP39 and polyhedrin (Polh) proteins at 72 h post-infection. Polypeptides from infected Tn368 cells were resolved on 12% SDS-polyacrylamide gels and transferred onto Immobilon-P (for VP39 protein) or nitrocellulose membranes (for polyhedrin). VP39 protein was probed using anti-BmNPV/AcMNPV VP39 polyclonal antibody and visualized with ECL Select Western Blotting Detection Reagent. Polyhedrin protein was probed with anti-BmNPV polyhedrin polyclonal antibody and visualized using a HRP Conjugate Substrate Kit. (**f**) Immunoblot analysis of luciferase and HCF-1 proteins in infected Tn368 cells. Luciferase and HCF-1 proteins were probed by anti-HA monoclonal antibody and visualized using ECL Western Blotting Detection Reagent and ECL Select Western Blotting Detection Reagent, respectively. In panels (**e**) and (**f**), polypeptides equivalent to 1 × 10^5^ cells were analyzed in each lane.

To determine whether HCF-1 promotes the productive infection of NPVs other than AcMNPV in Tn368 cells, transfection-infection experiments were performed using four different NPVs that are non-permissive in *T*. *ni* cells. Tn368 cells were first transfected with plasmids pFBD/hcf-1 and pFBD/luc, which express HCF-1 and luciferase proteins, respectively, under transcriptional control of the *Drosophila melanogaster* heat shock protein 70 (HSP70) gene promoter (Fig. 1a). At 24 h post-transfection, Tn368 cells were heat-shocked at 42 °C for 30 min and incubated for 6 h at 28 °C. The cells were then infected with HycuMNPV, OpMNPV, BmNPV and Lymantria dispar MNPV (LdMNPV), and examined for viral DNA, viral proteins, progeny budded viruses (BVs) and polyhedra. Microscopic examination at 72 h post-infection showed that polyhedra were only produced in a small number of HycuMNPV-infected Tn368 cells (Fig. 1b). However, progeny BV production by any of the examined NPVs was not detected (Fig. 1c). Although BV production was not observed, viral DNA synthesis was promoted by HCF-1 protein in Tn368 cells infected with HycuMNPV, OpMNPV and BmNPV (Fig. 1d). Notably, the production of major capsid protein VP39 and polyhedrin (matrix protein of polyhedra) were observed clearly only in HycuMNPV-infected Tn368 cells (Fig. 1e). In luciferase-expressing Tn368 cells, no significant increases in viral DNA, VP39 protein, polyhedrin, progeny BVs or polyhedra were detected following infection with any of the examined NPVs (Fig. 1b–e). Immunoblot analysis showed that substantial amounts of luciferase and HCF-1 proteins were expressed in the plasmid-transfected and virus-infected Tn368 cells (Fig. 1f).

### Construction and characterization of HycuMNPV bacmid (HycuBac) in SpIm cells

**Figure 2:**
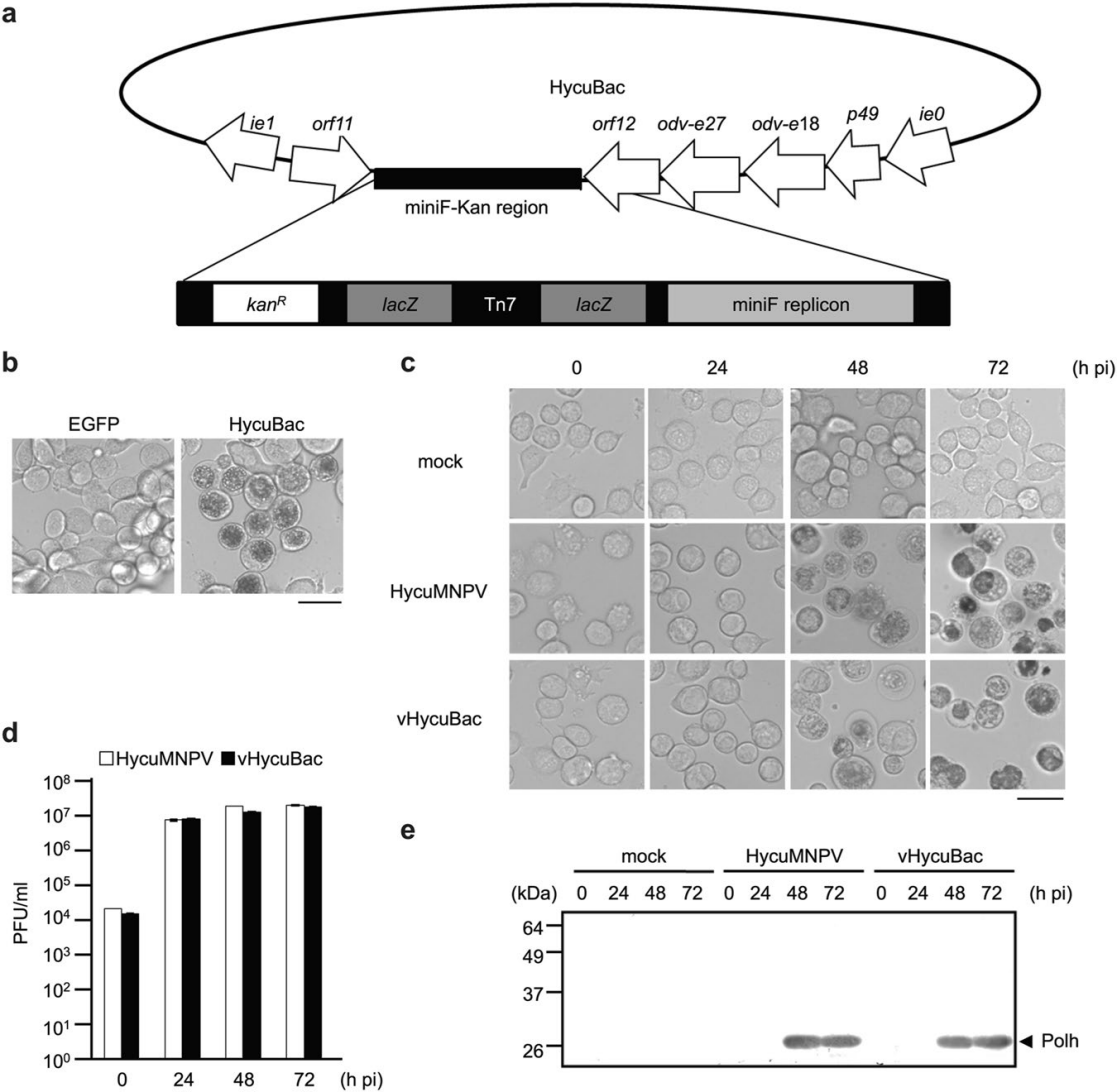
Characterization of HycuMNPV bacmid (HycuBac) in SpIm cells. (**a**) Schematic representation of HycuBac. (**b**) Microscopic images of SpIm cells transfected with pIE1-2/Egfp and HycuBac. SpIm cells were transfected with 2 μg of pIE1-2/Egfp, which expresses EGFP protein, or HycuBac and examined at 120 h post-transfection. Scale bar, 50 μm. (**c**) Microscopic images of virus-infected SpIm cells. mock, Mock-infected SpIm cells. Scale bar, 50 μm. (**d**) BV yields in virus-infected SpIm cells. BV titers were determined by a plaque assay using SpIm cells. The vertical bars represent the standard deviations of the averages from three determinations. (**e**) Immunoblot analysis of polyhedrin (Polh) protein in virus-infected SpIm cells. Polyhedrin was probed using anti-BmNPV polyhedrin polyclonal antibody and visualized with a HRP Conjugate Substrate Kit. mock, Mock-infected SpIm cells. Polypeptides equivalent to 1 × 10^5^ cells were analyzed in each lane. In (**c**), (**d**) and (**e**), SpIm cells were infected with HycuMNPV or HycuMNPV derived from HycuBac-transfected SpIm cells (vHycuBac) at an MOI of 10 and examined at 0, 24, 48 and 72 h post-infection (pi).

The results of the transfection-infection experiments presented in Fig. 1 suggested that HCF-1 protein promotes productive HycuMNPV replication in non-permissive Tn368 cells. To confirm this speculation, we constructed and characterized a HycuMNPV bacmid (HycuBac; Fig. 2a) in SpIm cells, which are permissive for HycuMNPV. HycuBac DNA or a plasmid that expresses EGFP protein was transfected into SpIm cells. At 72 h post-transfection, a number of polyhedra were found only in HycuBac DNA-transfected SpIm cells (Fig. 2b). Progeny BVs (vHycuBac) from HycuBac DNA-transfected SpIm cells and those of parental HycuMNPV were used to infect SpIm cells to compare the kinetics of polyhedra production, progeny BV yield and polyhedrin expression. All measured kinetic parameters of vHycuBac were comparable to those of parental HycuMNPV in infected SpIm cells up to 72 h post-infection (Fig. 2c–e).

### Construction and characterization of recombinant HycuBac harboring *hcf-1* or the *luciferase* gene in SpIm cells

**Figure 3:**
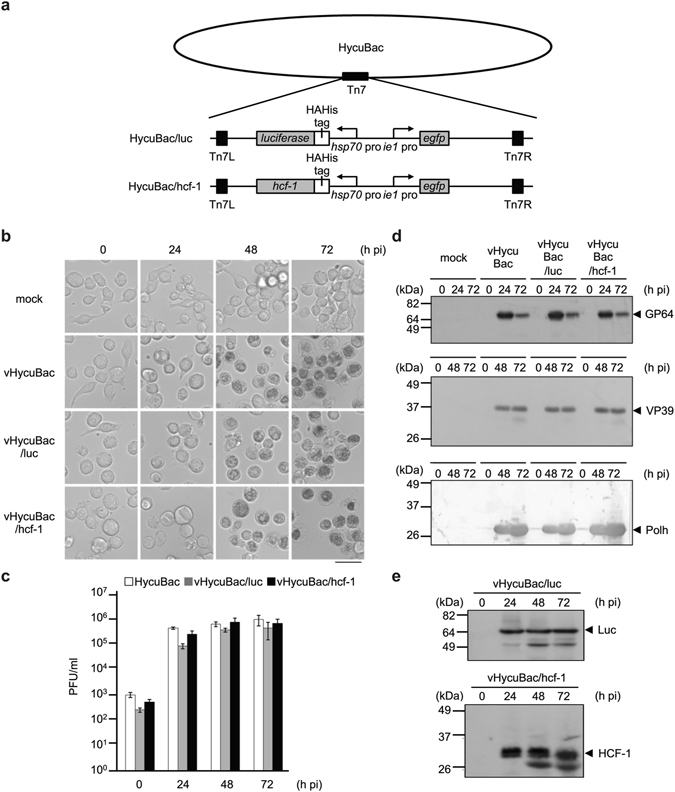
Characterization of recombinant HycuBac harboring hcf-1 (HycuBac/hcf-1) or luciferase gene (HycuBac/luc) in SpIm cells. SpIm cells were infected with vHycuBac or BVs derived from recombinant HycuBac/hcf-1 (vHycuBac/hcf-1) or HycuBac/luc (vHycuBac/luc) at an MOI of 5 and examined at 0, 24, 48 and 72 h post-infection (pi). (**a**) Schematic representation of HycuBac/luc and HycuBac/hcf-1. (**b**) Microscopic images of virus-infected SpIm cells. mock, Mock-infected SpIm cells. Scale bar, 50 μm. (**c**) BV yields in virus-infected SpIm cells. BV titers were determined by a plaque assay using SpIm cells. The vertical bars represent the standard deviations of the averages from three determinations. (**d**) Immunoblot analysis of GP64, VP39 and polyhedrin (Polh) proteins in virus-infected SpIm cells. GP64 was probed with anti-GP64 monoclonal antibody and visualized using ECL Western Blotting Detection Reagent. mock, Mock-infected SpIm cells. For details on the detection and visualization of the VP39 and polyhedron proteins, see the legend of Fig. 1e. (**e**) Immunoblot analysis of luciferase and HCF-1 proteins in infected SpIm cells. For details on the detection and visualization of luciferase and HCF-1, see the legend of Fig. 1f. In panels (**d**) and (**e**), polypeptides equivalent to 1 × 10^5^ cells were analyzed in each lane.

As HycuBac was shown to produce progeny BVs in SpIm cells with biological activity comparable to those of parental HycuMNPV, we constructed recombinant HycuBac harboring *hcf-1* (HycuBac/hcf-1) or the *luciferase* gene (HycuBac/luc) (Fig. 3a) to determine whether HCF-1 protein promoted HycuMNPV replication in Tn368 cells. The two types of progeny BVs, vHycuBac/hcf-1 and vHycuBac/luc, were obtained from SpIm cells transfected with HycuBac/hcf-1 and HycuBac/luc, respectively, and were then used to infect SpIm cells. No significant kinetic differences were observed between vHycuBac/hcf-1 and vHycuBac/luc with respect to polyhedra production, BV yield, and syntheses of envelope fusion protein GP64, major capsid protein VP39 and polyhedrin until 72 h post-infection (Fig. 3b–d). The luciferase and HCF-1 proteins were expressed at high levels in SpIm cells following infection with vHycuBac/hcf-1 and vHycuBac/luc, respectively (Fig. 3e).

### Recombinant HycuMNPV harboring *hcf-1* (vHycuBac/hcf-1) replicates in Tn368 cells

**Figure 4:**
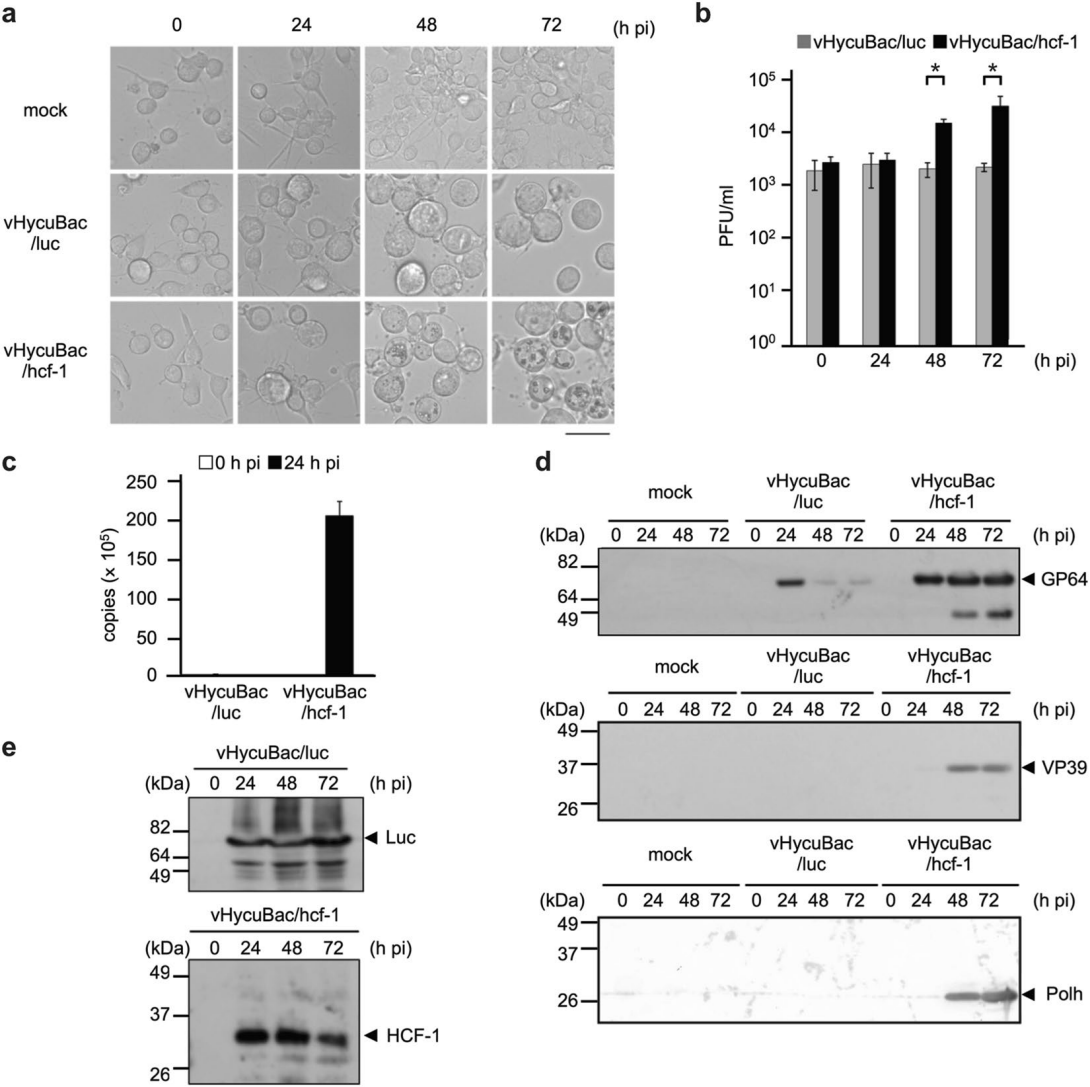
HycuMNPV harboring the hcf-1 gene replicates in non-permissive Tn368 cells. Tn368 cells were infected with vHycuBac, vHycuBac/hcf-1 or vHycuBac/luc at an MOI of 5 and examined at 0, 24, 48 and 72 h post-infection (pi). (**a**) Microscopic images of virus-infected Tn368 cells. mock, Mock-infected Tn368 cells. Scale bar, 50 μm. (**b**) BV yields in virus-infected Tn368 cells. BV titers were determined by a plaque assay using SpIm cells. The vertical bars represent the standard deviations of the averages from three determinations. Asterisks (*) indicate statistical significance (P < 0.05) in a Student t-test comparing vHycuBac/hcf-1 with vHycuBac/luc (P values = 0.0011 (48 h pi) and 0.0399 (72 h pi). (**c**) Viral DNA production in virus-infected Tn368 cells at 24 h post-infection. Viral DNA was quantified by qPCR. The vertical bars represent the standard deviations of the averages from three determinations. (**d**) Immunoblot analysis of GP64, VP39 and polyhedrin (Polh) proteins. mock, Mock-infected Tn368 cells. For details on the detection and visualization of GP64, VP39 and polyhedrin (Polh), see the legend of Fig. 3d. (**e**) Immunoblot analysis of luciferase and HCF-1 proteins in infected Tn368 cells. For details on the detection and visualization of luciferase and HCF-1, see the legend of Fig. 1f. In panels (**d**) and (**e**), polypeptides equivalent to 1 × 10^5^ cells were analyzed in each lane.

As no significant differences in the biological properties of vHycuBac/hcf-1, vHycuBac/luc and vHycuBac in SpIm cells were detected (Fig. 3), Tn368 cells were infected with vHycuBac/hcf-1 to determine whether HCF-1 protein expressed from the HycuMNPV genome promoted HycuMNPV replication in Tn368 cells. Microscopic examination showed that polyhedra were first produced at 48 h post-infection and that the number of cells with polyhedra had markedly increased by 72 h post-infection (Fig. 4a). BV production in vHycuBac/hcf-1-infected Tn368 cells increased continuously from 48 to 72 h post-infection (Fig. 4b) and the amount of viral DNA increased strikingly at 24 h post-infection (Fig. 4c). Envelope fusion protein GP64 accumulated substantially in vHycuBac/hcf-1-infected Tn368 cells from 24 h post-infection onwards (Fig. 4d) and VP39 and polyhedrin proteins were clearly detected at 48 h post-infection and remained detectable at 72 h post-infection in vHycuBac/hcf-1-infected Tn368 cells (Fig. 4d). In contrast, no significant production of polyhedra, BVs, viral DNA, VP39 protein or polyhedrin was observed in vHycuBac/luc-infected Tn368 cells (Fig. 4a–d). The GP64 band that was detected temporarily at 24 h post-infection in vHycuBac/luc-infected Tn368 cells (Fig. 4d) represents GP64 protein expressed through the *gp64* early promoter, which is occasionally observed upon infection with group I alphabaculoviruses even when inoculated viruses are unable to replicate productively. Immunoblot analysis showed that the luciferase and HCF-1 proteins were expressed at high levels from 24 to 72 h post-infection (Fig. 4e).

## Discussion

The *hcf-1* gene is required for the efficient replication of AcMNPV in Tn368 cells, but is dispensable for the productive infection of Sf21 cells^14, 15^, indicating that the HCF-1 functions specifically in Tn368 cells to expand the host range of AcMNPV. In the present study, we examined whether HCF-1 also promotes the replication of HycuMNPV, OpMNPV, BmNPV and LdMNPV in non-permissible Tn368 cells, and found that HycuMNPV harboring the *hcf-1* gene replicates in Tn368 cells, producing substantial amounts of BVs and polyhedra. These findings demonstrate that expansion of the AcMNPV host range to Tn368 cells by *hcf-1* can also be extended to other NPVs, suggesting that HCF-1 is an essential viral factor for productive NPV infection of Tn368 cells.

The *hcf-1* gene of AcMNPV is one of the *lef* (late expression factor) genes, which is essential for viral DNA replication and expression of late genes in transient assays in Tn368, but not Sf21, cells^14^. In AcMNPV-infected Tn368 cells, the HCF-1 protein is synthesized early during infection and localizes in punctuate nuclear structures^17, 18^. Recombinant AcMNPV defective in functional *hcf-1* gene replicates normally in Sf21 cells, but exhibits various mutant phenotypes in Tn368 cells, including defective viral DNA replication and late gene transcription, and complete cessation of cellular and viral protein syntheses^15^. These results collectively imply that the HCF-1 protein is involved in viral DNA replication and late gene expression, as well as cellular protein synthesis, in AcMNPV-infected Tn368 cells. However, the present results also showed that although OpMNPV and BmNPV DNAs were synthesized successfully in HCF-1-expressing Tn368 cells, major capsid protein VP39 and polyhedrin were not detected, and neither BVs nor polyhedra were produced. Quantitative qRT-PCR analysis revealed that transcripts from the *vp39* genes of HycuMNPV and OpMNPV, but not those of BmNPV or LdMNPV, increased markedly at 48 h post-infection in HCF-1-expressing Tn368 cells (Supplementary Figure S1). As no *vp39* gene transcripts were detected in Tn368 cells infected with BmNPV or LdMNPV, the *hcf-1* gene appears to promote viral late gene transcription in an NPV species-specific manner in Tn368 cells. In addition, because the OpMNPV *vp39* gene was transcribed at high levels in HCF-1-expressing Tn368 cells, recombinant OpMNPV harboring *hcf-1* may also be capable of replicating productively in non-permissive Tn368 cells.

A similar cell-line specific viral factor to HCF-1, designated as HRF-1, was identified in LdMNPV and functions specifically in Ld652Y cells derived from *L*. *dispar* ^13, 16^. The *hrf-1* gene was shown to rescue the global translation arrest of Ld652Y cells caused by AcMNPV infection and promotes productive AcMNPV replication in Ld652Y cells^13, 16^. We previously demonstrated that HRF-1 promotes the replication of BmNPV, HycuMNPV, and Spodoptera exigua MNPV in non-permissive Ld652Y cells, indicating that the HRF-1 protein also functions to expand the host range of various NPVs to non-permissive Ld652Y cells^19, 20^.

The *hcf-1* and *hrf-1* genes are encoded by only a few members of the family *Baculoviridae*. Evidence suggests that the *hrf-1* gene was acquired quite recently by LdMNPV through horizontal gene transfer^21^, and that homologs of HRF-1 with low amino acid sequence identity are found exclusively in OpMNPV^13, 22^, which productively infects Ld652Y cells^23^, and Dasychira pudibunda NPV, which is closely related to OpMNPV^24^. Homologs of *hcf-1* have been identified in only four NPV species: three close relatives of AcMNPV (Rachiplusia ou MNPV, Plutella xylostella NPV and Thysanoplusia orichalcea MNPV), and Clanis bilineata NPV, which is more distantly related to AcMNPV^8, 25–27^, suggesting that the *hcf-1* gene of AcMNPV was also acquired by a gene acquisition event. Notably, despite marked differences in structure and mode of action in virus-infected cells, HCF-1 and HRF-1 proteins have convergent functional roles in the host range expansion of NPVs to non-permissive cell lines derived from *T*. *ni* and *L*. *dispar*, respectively. Taken together with previous findings for the *hrf-1* gene^19, 20^, the present study indicates that several NPVs contain genes that are acquired by gene acquisition events and contribute to evolution of baculoviruses in terms of the host range expansion.

## Methods

### Cells, viruses and virus infection

Tn368 cells derived from *Trichoplusia ni* ^28^ were maintained at 28 °C in TC100 medium (AppliChem) supplemented with 0.26% tryptose broth (Sigma) and 10% fetal bovine serum (FBS). FRI-SpIm (SpIm) cells derived from *Spilosoma imparilis* ^29^ were grown at 28 °C in MM medium^30^ supplemented with 3% FBS. The following clonal isolates of four NPVs were used in these experiments: BmNPV N9 (BmNPV) from the silkworm *B*. *mori* ^31^, HycuMNPV N9 (HycuMNPV) from the fall webworm *H*. *cunea* ^32^, LdMNPV A21-MPV (LdMNPV) from the gypsy moth *L*. *dispar* ^33^, and OpMNPV from the Douglass-fir tussock moth, *O*. *pseudotsugata* ^34^.

Tn368 and SpIm cells were infected with viruses at varying multiplicity of infection (MOI) during a 60-min incubation at room temperature, as described previously^35^. Infected Tn368 and SpIm cells were washed three times with TC100 and MM media, respectively, and used for analyses at the indicated time points. The time zero of infection was defined as the time when the viral inoculum was removed from the cell culture.

### Plasmid construction

Plasmids pFBD/hcf-1 and pFBD/luc, which express HCF-1 and luciferase, respectively, under control of the HSP70 promoter (cf., Fig. 1a), were constructed using an In-Fusion HD Cloning Kit (Clontech). Linearized pFBD/pIE1-2/Egfp^36^ was obtained by inverse PCR using primers P1 and P2 (see Supplementary Table S1 for the primers used in this study). The region containing the HSP70 promoter and N-terminal HA- and His-tagged *hcf-1* open reading frame (ORF) was PCR-amplified from pHSHAHishcf-1VI+^12^ using primers P3 and P4, which contained 15-nt extension sequences complementary to the ends of linearized pFBD/pIE1-2/Egfp. The amplified *hcf-1* ORF region was cloned into linearized pFBD/pIE1-2/Egfp using an In-Fusion HD Cloning Kit, yielding pFBD/pIE1-2/Egfp/pHS/HAHisHcf-1 (pFBD/hcf-1). Control plasmid pFBD/pIE1-2/Egfp/pHS/HAHisLuc (pFBD/luc) was constructed by replacing the *hcf-1* gene of pFBD/hcf-1 with the *luciferase* gene that was amplified by PCR from pGL (Promega) using primers P5 and P6. *hcf-1*-defective linearized pFBD/hcf-1 was obtained by inverse PCR using primers P7 and P2. pFBD/hcf-1 and pFBD/luc were used as expression plasmids and for recombinant HycuMNPV bacmid construction. The construction of pIE1-2/Egfp, which expresses the EGFP protein, was described previously^37^. The plasmid sequences were confirmed by nucleotide sequencing using a BigDye Terminal v3.1 Cycle Sequencing Ready Reaction Kit and ABI 3130 Genetic Analyzer (Applied Biosystems), as described previously^38^.

### Construction of HycuMNPV bacmids

HycuBac containing a miniF replicon, *LacZα* gene, Tn7 insertion site and kanamycin-resistance gene (miniF-Kan region) (cf., Fig. 2a) was constructed in SpIm cells by homologous recombination between HycuMNPV genomic DNA and the transfer vector pHycuIE1-miniFKan-IE0 containing a miniF-Kan region. To construct pHycuIE1-miniFKan-IE0, the *ie1-ie0* region was PCR-amplified from HycuMNPV genomic DNA using primers P8 and P9, and the obtained amplicon was ligated to *Eco*RV-digested pBlueScript KS+ (Stratagene), yielding pHycuIE1-IE0. For construction of pHycuIE1-miniFKan-IE0, pHycuIE1-IE0 was linearized by inverse PCR using primers P10 and P11. The miniF-Kan region was PCR-amplified from AcMNPV bacmid DNA (Invitrogen) using primers P12 and P13, which contained 15-nt extension sequences complementary to the ends of linearized pHycuIE1-IE0. The obtained amplicon was cloned into linearized pHycuIE1-IE0 using an In-Fusion HD Cloning Kit, yielding pHycuIE1-miniFKan-IE0.

pHycuIE1-miniFKan-IE0 was co-transfected with HycuMNPV genomic DNA into SpIm cells using Lipofectin (Invitrogen), as described previously^39, 40^. Viral DNA was extracted from BVs and electroporated into DH10B *Escherichia coli* cells using a MicroPulser (BioRad). Positive transformants were selected on LB agar containing kanamycin (50 μg/ml), IPTG (24 μg/ml) and X-Gal (60 μg/ml). HycuBac DNA was isolated from *Lac*Z-positive and kanamycin-resistant colonies using a Qiagen Plasmid Midi Kit (Qiagen). HycuBac was then transfected into SpIm cells and culture medium containing vHycuBac BVs was collected at 168 h post-transfection. Working stocks of vHycuBac were amplified in SpIm cells.

Recombinant HycuBac harboring *hcf-1* (HycuBac/hcf-1) or *luciferase* gene (HycuBac/luc) (cf., Fig. 3a) was constructed through Tn7-mediated transposition in DH10B cells. DH10B cells carrying both HycuBac and the helper plasmid pMON7124 (Invitrogen) were transformed with pFBD/hcf-1 or pFBD/luc. The cells were incubated at 37 °C for 5 h and positive transformants were then selected on LB agar containing kanamycin (50 μg/ml), tetracycline (50 μg/ml), gentamicin (10 μg/ml), IPTG (24 μg/ml) and X-Gal (60 μg/ml). HycuBac/hcf-1 and HycuBac/luc DNA was purified from *LacZ*-negative colonies. The presence of *hcf-1* and *luciferase* with the correct sequence and structure in HycuBac/hcf-1 and HycuBac/luc DNAs, respectively, was verified by PCR analysis and DNA sequencing. BV stocks from HycuBac/hcf-1 (vHycuBac/hcf-1) and HycuBac/luc (vHycuBac/luc) were prepared as described above for vHycuBac.

### Immunoblot analysis

Immunoblot analyses of viral structural proteins, polyhedrin, and hemagglutinin (HA)-tagged HCF-1 and luciferase proteins were performed as described previously^37, 41^. Briefly, polypeptides from virus- and mock-infected cells were resolved on 12% SDS-polyacrylamide gels and blotted onto Immobilon-P (Millipore) or nitrocellulose membranes (Advantec Toyo) using a Trans-Blot Turbo system (Bio-Rad). GP64 and HA-tagged proteins (HCF-1 and luciferase) were probed with monoclonal antibodies against AcMNPV GP64 (Clontech) and HA (HA. 11; Babco), respectively, and were then reacted with horseradish peroxidase (HRP)-conjugated goat anti-mouse IgG antibody (Zymed Laboratories). VP39 and polyhedrin proteins were probed with polyclonal antibodies against BmNPV/AcMNPV VP39^42^ and BmNPV polyhedrin proteins^35^, respectively, and were then reacted with HRP-conjugated goat anti-rabbit IgG antibody (Zymed Laboratories). Positive signals were visualized using ECL Western Blotting Detection Reagent (GE Healthcare), ECL Select Western Blotting Detection Reagent (GE Healthcare) or an HRP Conjugate Substrate Kit (Bio-Rad).

### Quantitative PCR (qPCR) analysis

Quantification of *ie1* gene copies of HycuMNPV (*hycu-ie1*), OpMNPV (*op-ie1*), BmNPV (*bm-ie1*) and LdMNPV (*ld-ie1*) was performed by qPCR analysis, as described previously^36^. Briefly, total DNAs were extracted from virus-infected Tn368 cells using a QIAamp DNA Mini Kit (Qiagen) and subjected to qPCR analysis using a StepOnePlus Real-Time PCR system (Applied Biosystems) with the gene-specific probes Pb-1, Pb-2, Pb-3 and Pb-4 (Supplementary Table S2), and gene-specific primers P14 and P15, P16 and P17, P18 and P19, and P20 and P21 for *hycu-ie1*, *op-ie1*, *bm-ie1* and *ld-ie1*, respectively. The number of *ie-1* gene copies was determined using a standard curve generated from 10-fold dilutions of plasmids containing partial sequences of each *ie-1* ORF (pBS/hycuie1PS, pTOPO/opie1PS, pTOPO/bmie1PS and pTOPO/ldie1PS). The plasmids were constructed by cloning each partial sequence, which was amplified using the primers P22 and P23 for *hycu-ie1*, P24 and P25 for *op-ie1*, P26 and P27 for *bm-ie1*, and P28 and P29 for *ld-ie1*, into EcoRV-digested pBlueScript KS+ using DNA Ligation Kit Mighty Mix (Takara) or into pCR4-TOPO using a TOPO TA Cloning Kit (Invitrogen).

### Quantitative RT-PCR (qRT-PCR) analysis

Quantification of mRNAs expressed from the *vp39* genes of HycuMNPV (*hycu-vp39*), OpMNPV (*op-vp39*), BmNPV (*bm-vp39*) and LdMNPV (*ld-vp39*) was performed by qRT-PCR, as described previously^40^. Briefly, total RNAs were extracted from virus-infected Tn368 cells using a SYBR Green Cells-to-CT Kit (Ambion), treated with DNase I using an RNAqueous-4PCR Kit (Ambion) and reverse-transcribed to cDNAs using a SYBR Green Cells-to-CT Kit. The resultant cDNAs were subjected to qRT-PCR analysis using a StepOnePlus Real-Time PCR system with the gene-specific primers P30 and P31, P32 and P33, P34 and P35, and P36 and P37 for *hycu-vp39*, *op-vp39*, *bm-vp39* and *ld-vp39*, respectively. The number of each *vp39* mRNA was calculated using a standard curve generated from 10-fold dilutions of plasmids containing entire or partial sequences of each *vp39* ORF (pTOPO/hycuvp39, pTOPO/opvp39, pTOPO/bmvp39 and pTOPO/ldvp39PS). The plasmids were constructed by cloning each sequence using the primers P38 and P39 for *hycu-vp39*, P40 and P41 for *op-vp39*, P42 and P43 for *bm-vp39*, and P37 and P44 for *ld-vp39* into pCR4-TOPO using a TOPO TA Cloning Kit.

### Plaque assay

Plaque assays were performed using SpIm cells for HycuMNPV, BM-N cells for BmNPV and Ld652Y cells for OpMNPV and LdMNPV, as described previously^35^. Briefly, monolayer cultures of these cells (0.5 × 10^6^ cells) were prepared in 35 mm culture dishes (Falcon 3001) and inoculated with 200 μl of the virus suspensions to be titrated. After a 60 min adsorption period, the viral inoculum was replaced with 3 ml of 0.75% SeaPlaque agarose (FMC) in the medium appropriate for each cell line. The cultures were incubated at 28 °C for 5–6 days and formed plaques were counted under a microscope.

### Transfection

The transfection of SpIm and Tn368 cells with expression plasmids and bacmids was performed using Lipofectin (Invitrogen) and FuGENE (Promega), respectively, as described previously^39, 43^.

## Electronic supplementary material

Dataset 1
