# Supplementary material for: HCF-1 encoded by baculovirus AcMNPV is required for productive nucleopolyhedrovirus infection of non-permissive Tn368 cells
Source: Sci Rep. 2017 Jun 19;7:3807. doi: 10.1038/s41598-017-03710-z (PMC5476645; doi:10.1038/s41598-017-03710-z)
Supplement: Supplementary file 1 — Dataset 1 [file 41598_2017_3710_MOESM1_ESM.zip › Supplementary_tachibana.pdf]

Supplementary information

HCF-1 encoded by baculovirus AcMNPV is required for productive nucleopolyhedrovirus infection of non-permissive Tn368 cells

Ami Tachibana, Rina Hamajima, Moe Tomizaki, Takuya Kondo, Yoshie Nanba, Michihiro Kobayashi, Hayato Yamada, Motoko Ikeda*

Laboratory of Sericulture and Entomoresources, Graduate School of Bioagricultural Sciences, Nagoya University, Chikusa, Nagoya 464-8601, Japan.

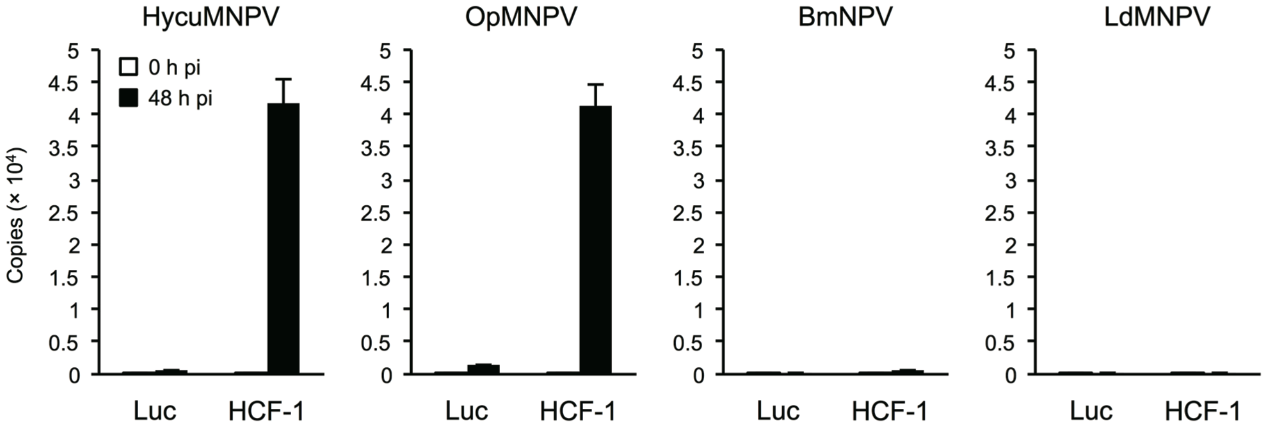

Supplementary Fig. S1. Transiently expressed HCF-1 protein promotes transcription of the viral late gene *vp39* of certain NPVs in non-permissive Tn368 cells. Luciferase-expressing (Luc) and HCF-1-expressing Tn368 cells (HCF-1) were infected with HycuMNPV, OpMNPV, BmNPV and LdMNPV. At 0 and 48 h post-infection (pi), total RNAs were extracted from infected cells using a Power SYBR Green Cells-to-CT Kit, treated with DNase I and then subjected to qRT-PCR analysis using gene-specific primers and SYBR Green Master Mix.

Supplementary Table S1. Nucleotide primers used in this study

Name	Sequence
P1	5'-CTCCATGATCTATTAATATTCCGG-3'
P2	5'-ATTCAAAGGCCTACGTCGAC-3'
P3	5'-TAATAGATCATGGAGTTAAGCAGCAGCCGTATTTATAAAG-3'
P4	5'-CGTAGGCCTTTGAATCCGGGTTAAACATTTATTAACTTG-3'
P5	5'-CATCATCACAGATCTGAAGACGCCAAAAACATAAAGAAAGG-3'
P6	5'-CGTAGGCCTTTGAATTTACACGGCGATCTTTCCG-3'
P7	5'-AGATCTGTGATGATGATGATGATGC-3'
P8	5’-TGCCAAGTTGTGCTTTCGCGCC-3’
P9	5’-GTGATGTCAGGCAACGTGGAGC-3’
P10	5’-ACAATTGTTACGGTTGTAGTTAAAATGAAACCG-3’
P11	5’-AAATAATCACATTTGTAAATCTAATTGTACCGGAC-3’
P12	5’-CAAATGTGATTATTTTTTTGCTTTGCCACGGAACG-3’
P13	5’-AACCGTAACAATTGTTTGCCGGGTCCCAGGAA-3’
P14	5'-AATGCAGTCTATGGGCGAAAAC-3'
P15	5'-CGGTCTTGGTACATTTGAAAAAGC-3'
P16	5'-TGACGACACGAGCCACAACT-3'
P17	5'-GTTAGCCGCTCGTCGTTGAC-3'
P18	5'-GGAGCCGACGGTAGTATCTG-3'
P19	5'-GAATTGACGCTTGCCAAAAAGT-3'
P20	5'-TCGCCACGTCGACAACAA-3'
P21	5'-CGAGATCATAAAGCGGCACTT-3'
P22	5'-GCCGTTTGAAGTGGTGTTTG-3'
P23	5'-CGCGATGTCCGTACCTGT-3'
P24	5'-TTGAGCCGGTGAAAAACGCG-3'
P25	5'-CGTCCACGCTAATGTCCTTG-3'
P26	5'-TCGACAACGGCTATTCAGAG-3'
P27	5'-CAGTCTCGGACATATGGTCGG-3'
P28	5'-AAGGTGACCTACGCGAACTG-3'
P29	5'-TGACCGAGTCCAGAGCCTG-3'
P30	5'-AACGTGGTGGAAAGCGTGTAC-3'
P31	5'-GCCACGCGTGTGCAATAC-3'
P32	5'-CAATTTGCCGGGCATGA-3'
P33	5'-AAATGCGATTGACTTCCAAACG-3'
P34	5'-CGACGCGTGCATAACATACAA-3'
P35	5'-GGTGGCTCTTGCAGATAAACC-3'
P36	5'-AACCGAACGAGCGCCATAC-3'
P37	5'-CAGATGCGCGCGTAATTG-3'
P38	5'-ATGGCACTAGTGTCACCCG-3'
P39	5'-TTACACTGCTTCTGCTGGTTG-3'
P40	5'-ATGGCGCTCGTGTCACC-3'
P41	5'-TTAAACAACGGGCGCTG-3'
P42	5'-ATGGCGCTAATGCCCGTG-3'
P43	5'-TTAGGCGGCTACACCTCC-3'
P44	5'-ATGGCACTTGTGAGCGG-3'

Sequences of extensions complementary to vector ends are underlined.

Supplementary Table S2. Oligonucleotide probes used in this study

Name	Sequence
Pb-1	5'-CAGGCATGTTGCTCAA-3'
Pb-2	5'-ATCGTCCTTCAGTTTGG-3'
Pb-3	5'-CTGCAGCAGCTTCA-3'
Pb-4	5'-ATGGTGCTGTCGATAGA-3'
